# Workplace smoking restrictions and support for smoking cessation in the USA: state, region, and overall trends from 2010–11 to 2014–15

**DOI:** 10.1017/jsc.2019.10

**Published:** 2019-07-22

**Authors:** Yujiao Mai, Trung Ha, Julia N. Soulakova

**Affiliations:** Burnett School of Biomedical Sciences, College of Medicine, University of Central Florida, 6900 Lake Nona Blvd., Orlando, FL 32827, USA

**Keywords:** Multiple logistic regression, national survey, smoking policy, worksite tobacco control

## Abstract

We discuss the most recent changes in smoking policies and support for smoking cessation offered to smokers at US workplaces. We used reports of employed adults (*n* = 112,008) regarding smoking restrictions and support for smoking cessation offered at their indoor workplaces from the 2010–11 and 2014–15 Tobacco Use Supplement-Current Population Survey. The percentage of adults who reported having workplace smoking restrictions was 94% in 2010–11 and 93% in 2014–15 (*P* = 0.001). There was a decrease in the Northeastern region (*P* < 0.001) and no significant changes in the other three US regions. The percentages decreased in Hawaii, New York, Oregon, Pennsylvania, and Tennessee and increased in Indiana, Nebraska, and Wyoming. The percentage of employees who reported having workplace support for smoking cessation increased from 24% to 29% (*P* < 0.001), which was uniform across all US regions but differed across the US states. The percentages decreased in Hawaii and increased in the majority of states. Analysis of smokers’ reports (versus all reports) resulted in lower percentages of workplaces with smoking restrictions and support for smoking cessation. It is essential to further enhance support for smoking cessation offered to smokers at US workplaces.

## Introduction

Smoking and exposure to second-hand smoke have been linked to increased risks for lung cancer, heart disease, respiratory illness, and many other health problems (Brownson, Eriksen, Davis, & Warner, 1997; He et al., 1999; Janerich et al., 1990; Lam et al., 2005; U.S. Department of Health and Human Services, 2006; 2010, 2014). From 1964 to 2014, smoking and exposure to second-hand smoke in the USA caused more than 20 million premature deaths and accounted for about $300 billion annual expenditure due to medical care and productivity loss (U.S. Department of Health and Human Services, 2014).

For employed adults, smoking policies at the workplace are the primary means for reducing tobacco consumption and exposure to second-hand smoke (Borland, Chapman, Owen, & Hill, 1990; Brownson, Hopkins, & Wakefield, 2002; Cheng, Liu, Gonzalez, & Glantz, 2017). Specifically, two meta-analyses of more than 20 studies each indicated that smoking prevalence and nicotine dependence is lower among employees who work at smoke-free workplaces relative to workplaces that are not smoke-free (Fichtenberg & Glantz, 2002; Hopkins et al., 2010). In the USA, the majority of workplaces currently have some type of smoking restrictions. For example, the overall rate of workplaces with smoking restrictions in indoor areas was about 79% in the period from 2001 to 2010 (Cheng et al., 2017).

Many studies have detected discrepancies in the percentages of workplaces with smoking policies using reports provided by employees. These percentages varied significantly by employee’s age, sex, race/ethnicity, education, occupation, income, and smoking status (Delnevo, Hrywna, & Jane Lewis, 2004; Gerlach, Shopland, Hartman, Gibson, & Pechacek, 1997). Specifically, the percentage of employed individuals who had smoking restrictions at their workplaces was higher among older than younger employees (Gerlach et al., 1997), women than men, non-Hispanic Whites and Asian/Pacific Islanders than Hispanics and non-Hispanic Blacks (Gerlach et al., 1997), higher- than lower-educated employees (Burns, Shanks, Major, Gower, & Shopland, 2000; Delnevo et al., 2004), employees with higher household income than employees with lower household income (Burns et al., 2000; Delnevo et al., 2004), white-collar workers than the service and blue-collar workers (Alexander, Crawford, & Mendiondo, 2010; Gerlach et al., 1997), and non-smokers than smokers (Gerlach et al., 1997).

In the period from 1985 to 2006, workplace smoking policies became more prevalent in the USA (Burns et al., 2000; Cheng et al., 2017; Delnevo et al., 2004; Gerlach et al., 1997; Halbesleben & Wheeler, 2010). Specifically, the percentage of workplaces with smoking restrictions in indoor areas was 38% in 1985, 82% in 1992–93, and 86% in 1995–96 (Burns et al., 2000; Evans, Farrelly, & Montgomery, 1999; Farrelly, Evans, & Sfekas, 1999; Gerlach et al., 1997). Likewise, the percentage of smoke-free indoor workplaces was about 3% in 1986 and 74% in 2006; however, there was no significant difference in the rates from 2000 to 2006 (Halbesleben & Wheeler, 2010). In addition, discrepancies across the states and regions in the percentages of workplaces with smoking restrictions have been noted (Burns et al., 2000; Delnevo et al., 2004; Halbesleben & Wheeler, 2010; Shavers et al., 2006; Shopland, Gerlach, Burns, Hartman, & Gibson, 2001). For example, in 1995–96, the percentage of workplaces with smoking restrictions varied from 75% in Mississippi to 95% in Maryland (Burns et al., 2000).

Despite the significant contributions of the studies mentioned above, there is a lack of research addressing the recent progress in the implementation of workplace smoking policies made in the USA. Additionally, there is a lack of knowledge regarding the support for smoking cessation offered at workplaces; e.g., is it common that workplaces offer insurance coverage for a smoking cessation intervention or treatment to employee-smokers? We found only one relevant study estimating that in 2004, only 19% of worksites offered support for smoking cessation (Linnan et al., 2008). Our research aims were:

1. To estimate the changes in the percentage of workplaces with smoking restrictions within states, regions, and overall from 2010–11 to 2014–15, and
2. To estimate the changes in the percentage of workplaces with support for smoking cessation within states, regions, and overall from 2010–11 to 2014–15.

When addressing the research aims, we also assessed the disparities associated with employees’ sociodemographic characteristics. Because smokers are potentially better aware of smoking restrictions and support for smoking cessation offered at their workplace, we examined discrepancies in the percentages among smokers and all employees (smokers and nonsmokers combined).

## Methods

### Survey data

The Tobacco Use Supplement-Current Population Survey (TUS-CPS) is administered to gather information regarding tobacco use, including cigarette smoking, attitudes toward smoking bans, smoking restrictions at workplaces, and many other events and behaviors related to tobacco use and cessation (Alexander et al., 2010; Cheng et al., 2017; Mills, Messer, Gilpin, & Pierce, 2009; Shavers et al., 2006; Shopland et al., 2001). The US Census Bureau started administering the TUS-CPS in 1992. The Bureau publishes the de-identified TUS-CPS data online for public use. We pooled the TUS-CPS data from two survey periods, 2010–11 and 2014–15, where each period consisted of three monthly waves conducted in August 2010, January 2011, and May 2011, and July 2014, January 2015, and May 2015. When pooling the data, we created an index variable to define the survey period, merged all common variables, and adjusted the weights; e.g., the weights for each monthly wave were divided by 6. We identified 112,008 employed adults (18 years old and older) who (a) resided and worked in the same state, (b) worked indoors, (c) were not self-employed, and (d) were not working at their or someone else’s home. Table 1 presents the sample characteristics and that 16,456 respondents were smokers. Supplementary Table presents state-level sample sizes for all employees and smokers only.

### Study measures

The two primary measures were:

- Smoking restrictions at the workplace (yes – smoking restrictions exist at the workplace, no – there are no smoking restrictions at the workplace) and
- Support for smoking cessation offered at the workplace (yes – support for smoking cessation is offered at the workplace, no – there is no support for smoking cessation offered at the workplace).

These measures were defined, respectively, using ‘Yes’ and ‘No’ responses to the survey items, ‘Is smoking restricted in any way at your place of work?’ and ‘Within the past 12 months, has your employer offered any stop smoking program or any other help to employees who want to quit smoking?’ We note that both measures are based on the employees’ reports and thus the corresponding percentages refer to the percentages of employees who reported that there were smoking restrictions at their workplace and there was support for smoking cessation offered at their workplace. Because some respondents could actually work at the same workplaces, the percentages of employees who reported some characteristics of their workplace might be different from the actual percentages of workplaces with these characteristics. However, for the convenience of presentation, we omit the detailed wording in some places in the paper, e.g., in the section ‘Statistical analysis.’

Table 1 lists all explanatory measures. We note that the state (region) of residence was identical to the state (region) of employment.

### Statistical analysis

To adjust for the complex design of the TUS-CPS, we incorporated the main and replicate weights in all analyses and used Balanced Repeated Replications with Fay correction for variance estimation (U.S. Department of Commerce, Census Bureau, 2016; Wolter, 2007). To perform computing and modeling, we used Survey Package in SAS/STAT®13.1 (SAS Institute Inc., 2013). The significance level was 5% for each aim. Statistical analyses were performed in the period from May to August of 2017.

The primary analyses incorporated two multiple logistic regression models: one for the *logit* of workplace smoking restrictions (Likelihood Ratio *χ*^2^ = 1, 123, 525, *df* = 25, *P* < 0.001) and the other for the *logit* of support for smoking cessation offered at the workplace (Likelihood Ratio *χ*^2^ = 3, 661, 859, *df* = 25, *P* < 0.001). Each model included the main effects (employee’s age, sex, race/ethnicity, marital status, highest level of education, metropolitan status of residence, region of residence, smoking status, survey mode and survey year) and the two-way interaction between the region and year (both *Ps* < 0.001). In the model for the *logit* of workplace smoking restrictions, all main effects except for metropolitan status of residence were significant (all *Ps* < 0.05). In the model for the *logit* of support for smoking restrictions at the workplace all main effects except for the survey mode were significant (all *Ps* < 0.05). The models were used to assess the over-time differences within each region.

To perform multiple comparisons for significant effects we used Bonferroni adjustments. For example, when assessing the over-time differences within each region, we computed each adjusted *p*-value as 4 times the original *P*-value. Similarly, we constructed 98.75% individual confidence intervals so that the simultaneous confidence band had a 95% confidence level.

To assess the over-time differences within each state, we constructed two-way contingency tables for associations between a primary measure and year (2010–11, 2014–15) stratified by state. In addition, we constructed similar tables using reports by smokers only. To assess the significance of these associations we used two-tailed Rao-Scott *χ*^2^ tests (Rao & Scott, 1984). We report the original two-tailed *P*-values and thus, these analyses should be viewed as individual within each state.

## Results

### Changes in percentage of workplaces with smoking restrictions within states, regions, and overall from 2010–11 to 2014–15

#### Trends within States

Table 2 illustrates the 2010–11 and 2014–15 percentages of employees who reported that smoking restrictions existed at their workplaces and significance of the difference within each state. Based on all reports, the following within-state differences were significant *(Ps* < 0.05): percentage increased in Indiana, Nebraska, and Wyoming and decreased in Hawaii, New York, Oregon, Pennsylvania, and Tennessee. The lowest 2014–15 percentages (90.0% or below) corresponded to Nevada, New Mexico, Mississippi, and Oklahoma. Based on smokers’ reports, the within-state differences were significant (*Ps* < 0.05) in District of Columbia, New York, Montana, and Wyoming: the percentages decreased in District of Columbia, New York, and Montana, and increased in Wyoming.

#### Trends within regions

**Fig. 1.**
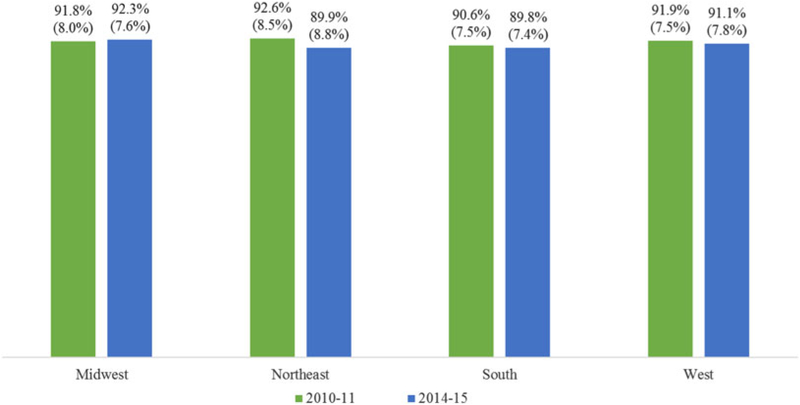
Model-assisted percentages (and standard errors) of workplaces with smoking restrictions; the over-time difference was significant only in the Northeastern region.

Among the four US regions, the significant over-time difference in the percentage of workplace smoking restrictions was detected in the Northeastern region only: the percentage was lower in 2014–15 than it was in 2010–11 (OR = 0.70, 98.75% CI = 0.58:0.86, adjusted *P* < 0.001). The over-time differences were not significant in the Midwestern, Southern, and Western regions. Figure 1 presents the percentages of workplaces with smoking restrictions for each region.

#### Overall trend

About 93.2% of US employees reported that there were smoking restrictions at their workplace in the period from 2010–11 to 2014–15. The percentage decreased significantly (*P* = 0.001) from 93.6% in 2010–11 to 92.9% in 2014–15. This was also supported by the model – the overall odds were lower in 2014–15 relative to 2010–11 (OR = 0.89, 95% CI = 0.83:0.95, *P* < 0.001). Among smokers, the overall percentage of those reporting that there were smoking restrictions at their workplaces was 91.5%; the over-time difference was not significant – 91.7% in 2010–11 and 91.2% in 2014–15.

#### Additional results

Table 3 presents results for level comparisons within sociodemographic factors (other than region of residence) and smoking status. The table illustrates disparities in the percentages of employees who reported having workplace smoking restrictions across employee’s age, sex, race/ethnicity, marital status, and highest level of education, as well as smoking status. Specifically, the percentage was lower among 18–24 year-old than 65+ year-old employees, and higher among 45–64 year-old employees than 65+ year-old employees; lower among men than women; lower among non-Hispanic Black, non-Hispanic Asian, and Hispanic than non-Hispanic White employees; higher among currently and ever married than never married employees; lower among less educated employees than those who had graduate education (or equivalent); and lower among smokers than nonsmokers. In addition, phone interviews corresponded to a higher percentage of workplaces with smoking restrictions in comparison to interviews conducted in-person (OR = 1.12, 95% CI = 1.06:1.20).

### Changes in percentage of workplaces offering support for smoking cessation within states, regions, and overall from 2010–11 to 2014–15

#### Trends within states

Table 2 illustrates the state-level percentages of employees who reported that support for smoking cessation was offered at their workplace. Based on all reports, the percentage increased in 32 states including the District of Columbia and decreased in Hawaii; the highest 2014–15 percentages (40.0% or more) corresponded to Oregon, Minnesota, Wisconsin, Maine, and Vermont; and the lowest 2014–15 percentages (20.0% or less) corresponded to Hawaii, and Louisiana. Based on smokers’ reports, the percentages increased in Alabama, Delaware, Missouri, New Mexico, Tennessee, Texas, and Virginia and did not decrease significantly in any US state.

#### Trends within regions

**Fig. 2.**
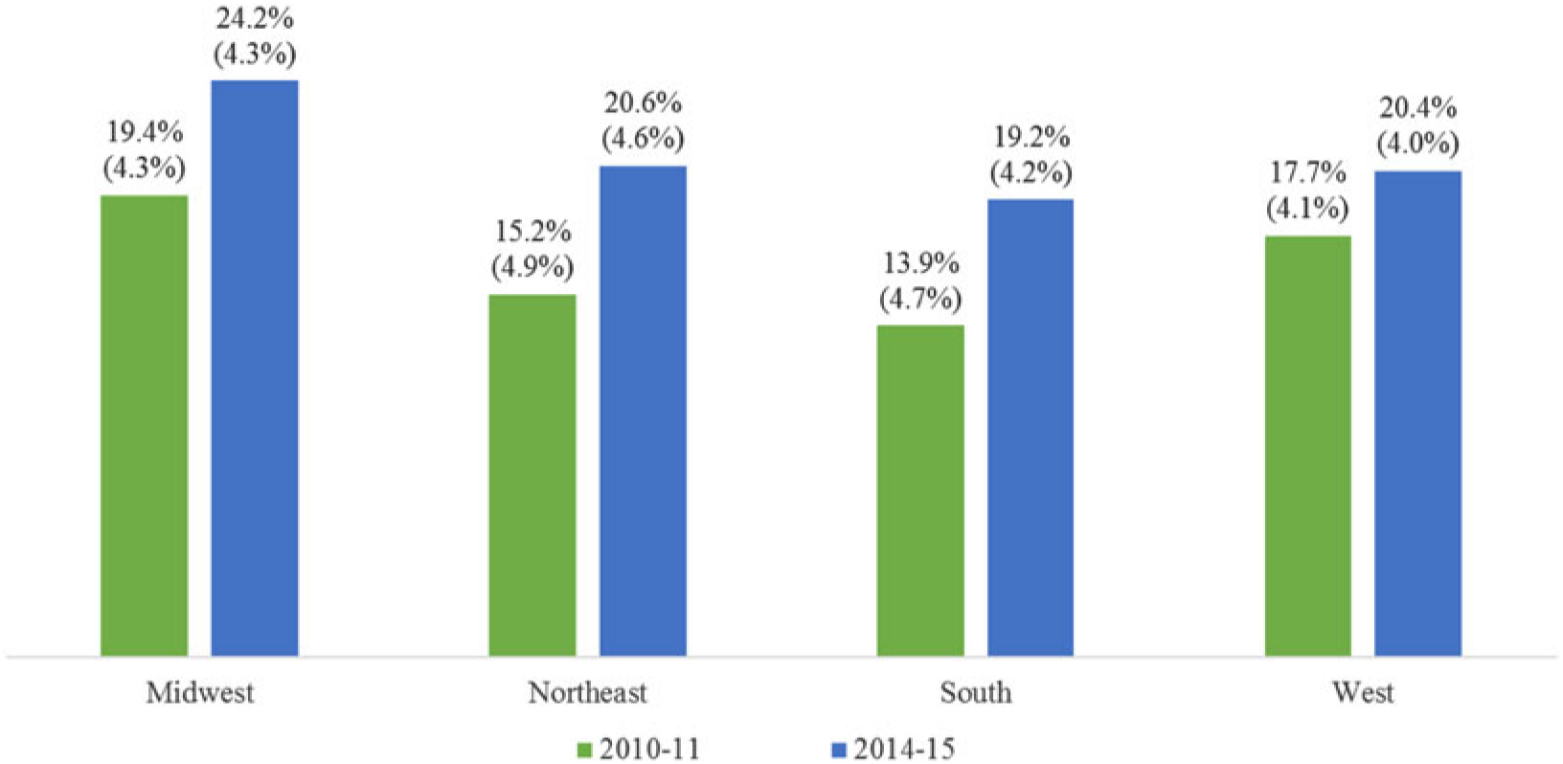
Model-assisted percentages (and standard errors) of workplaces with support for smoking cessation; the over-time differences were significant within each region.

Significant over-time differences in the percentages of employees who reported that support for smoking cessation was offered at their workplace were detected within all regions (adjusted *Ps* < 0.001). Specifically, the odds were higher in 2014–15 than in 2010–11 in the Midwest (OR = 1.32, 98.75% CI = 1.22:1.44), Northeast (OR = 1.44, 98.75% CI = 1.29:1.62), South (OR =1.47, 98.75% CI = 1.36:1.60), and West (OR = 1.19, 98.75% CI = 1.09:1.30). Figure 2 presents the corresponding percentages.

#### Overall trend

About 26.6% of employees reported that support for smoking cessation is offered at their workplace in the US in the period from 2010–11 to 2014–15. The percentage increased significantly (*P* < 0.001) from 23.7% in 2010–11 to 29.4% in 2014–15. This trend was also supported based on the model – the odds were higher in 2014–15 relative to 2010–11 (OR = 1.35, 95% CI = 1.29:1.42, *P* < 0.001). Based on smoker’s reports, about 22.4% of smokers reported that support for smoking cessation was offered at their workplace; this estimate refers to the overall percentage in the period from 2010–11 to 2014–15. The percentage increased significantly (*P* < 0.001) from 20.2% in 2010–11 to 24.8% in 2014–15.

#### Additional results

Table 3 illustrates that the percentages of employees who reported that there was workplace support for smoking cessation varied across employees’ sociodemographic characteristics. The percentage was lower among 18–24 year-old than 65+ year-old employees, and higher among 25–44 and 45–64 year-old employees than 65+ year-old employees; higher among men than women; lower among non-Hispanic Asian and Hispanic than non-Hispanic White employees, and higher among non-Hispanic Multiracial than non-Hispanic White employees; higher among currently and ever married than never married employees; lower among less educated employees than those who had graduate education (or equivalent); higher among employees residing in a metropolitan area than a non-metropolitan area; and lower among smokers than nonsmokers.

## Discussion

### Main findings

About 93% of US employees reported that smoking restrictions existed at their workplace in the period from 2010–11 to 2014–15. There was a slight over-time decrease (about 1%) in the percentage; however, in comparison to the 1995–96 rate of 86% (Burns et al., 2000), our study points to a substantial improvement in the implementation of workplace smoking restrictions in the past two decades. The over-time decrease was significant within the Northeastern region only but the difference of 2.7% might not be of practical importance given that the 2014–15 percentage is still high (about 90%). The over-time differences were not significant within the three other regions but were different across the US states: the positive trends (that were significant within the state) were observed in Indiana, Nebraska, and Wyoming, while the negative trends were observed in Hawaii, New York, Oregon, Pennsylvania, and Tennessee.

The overall percentage of US employees who reported that there is support for smoking cessation at their workplace was about 27% in the period from 2010–11 to 2014–15. The percentage increased significantly from 24% in 2010–11 to 29% in 2014–15. This positive trend was significant within each US region as well as within the majority of states. The negative trend was observed only in Hawaii. Therefore, the study indicates important positive changes in support for smoking cessation at US workplaces.

### Secondary findings

Surprisingly, the percentages estimated using smokers’ reports were considerably lower than the corresponding percentages estimated using all reports for both measures. For example, the 2010–11 percentage of smokers who reported having support for smoking cessation at their workplaces was about 20% in comparison to 24% based on reports of all employees (smokers and nonsmokers combined) and the 2014–15 percentage of smokers who reported having support for smoking cessation at their workplaces was about 25% in comparison to 29% based on reports of all employees. Some of these discrepancies projected to the states as well. For example, based on all reports, the positive trend in support for smoking cessation was observed for the majority of states, however, based on smokers’ reports, the positive trend was observed for seven states only.

There are several possible reasons for observing the lower percentages based on smokers’ reports relative to all employees’ reports. One of the reasons is that smokers and nonsmokers worked at workplaces that indeed differed (on average) in terms of these two measures, that is, occupation is a confounder. In addition, smokers and nonsmokers could have different perceptions regarding existing smoking restrictions and support for smoking cessation and these perceptions could affect their responses. For example, if a smoker is aware of existing support for smoking cessation but perceives it insufficient (or not helpful) then he/she might intentionally report ‘there is no support for smoking cessation at my workplace.’ This reasoning is in line with the prior research findings regarding response bias when reporting smoking behaviors (Johnson & Mott, 2001; Soulakova, Bright, & Crockett, 2015; Soulakova, Davis, Hartman, & Gibson, 2009).

The study also pointed to some common (for both measures) differences in the reported percentages associated with employees’ age, race/ethnicity, marital status, and education:

- lower (higher) percentages corresponded to 18–24 (45–64) year-old employees relative to 65+ year-old employees,
- lower percentages corresponded to non-Hispanic Asian and Hispanic employees relative to non-Hispanic White employees,
- higher percentages corresponded to ever-married employees than never married employees, and
- lower percentages corresponded to less-educated employees relative to employees with at least some graduate education or equivalent.

These results appear to be consistent with results of prior studies. However, the definitions of the primary measures (workplace smoking restrictions and support for smoking cessation) did not match precisely between this and prior studies, prohibiting direct comparisons (Delnevo et al., 2004; Gerlach et al., 1997).

### Limitations

The study has several limitations. First, due to the cross-sectional nature of the TUS–CPS, relationships addressed in this paper should not be treated as causal. Second, the surveys were conducted in 2010–11 and 2014–15 and thus, year-specific trends (e.g., from 2012 to 2013) could not be examined using the TUS-CPS data. Third, the defined measure of workplace smoking restrictions referring to indoor work areas was rather broad, and a study of a more specific measure, e.g., a smoke-free workplace, could be more informative. In addition, the data were self-reported and thus may be affected by the response bias mentioned above (Cohen & Conway, 2007; Johnson & Schultz, 2005). Finally, the study did not consider smoking rules among those employees who worked outdoors, were self-employed and/or worked from home. Therefore, the study results should not be generalized to these types of employment and workplaces.

### Future research

State laws for indoor tobacco use at workplaces vary drastically by states, e.g., in 2019 there were 43 US states and the District of Columbia that had local laws requiring all non-hospitality workplaces to be smoke-free while the other seven US states did not have such laws regarding smoking restrictions at the workplace (American Nonsmokers’ Rights Foundation, 2019). Future research should examine the impact of state policies regarding indoor tobacco use at workplaces on worksite smoking restrictions. In addition, studies that utilize reports of both employers and employees from the same workplace, would help evaluate possible differences in perceptions associated with the occupation type and assess employees’ awareness of the existing smoking policies and/or support for smoking cessation offered at their workplace.

## Conclusions

The Affordable Care Act (ACA) was signed in 2010 and many ACA policies, including federal guidelines for medical insurances to cover some types of smoking cessation treatments, have been implemented in the past decade (Blumenthal & Collins, 2014; Sommers, Gunja, Finegold, & Musco, 2015). Therefore, substantial improvements in workplace support for smoking cessation, observed in our study, could be (at least in part) the result of the ACA. However, the study also indicated that these improvements varied by state: while positive changes were observed within some states, there were no substantial changes or even negative changes within the other states. We present the within-state estimates for the percentages of workplaces with smoking restrictions and workplaces offering support for smoking cessation (estimated based on employees’ reports, all and smokers only). These estimates could be informative for state policymakers, healthcare professionals, and researchers who aim to promote smoking cessation and smoke-free environments.

## Supplementary Material

1

## Figures and Tables

**Table 1.** Sample Characteristics (*n* = 112,008)

Characteristic	Count	Percent	Characteristic	Count	Percent
Age			Highest level of education		
18–24	9,796	14.3	Below high school	5,899	6.0
25–44	50,691	45.0	High school/equivalent	26,637	23.8
45–64	46,456	37.2	Some/completed college	62,560	55.8
65+	5,065	3.6	Above college	16,912	14.4
Sex			Metropolitan status of residence		
Male	45,262	44.9	Metropolitan	90,509	87.0
Female	66,746	55.1	Non-metropolitan	21,499	13.0
Race/ethnicity			Region of residence		
Non-Hispanic (NH) White	81,869	66.5	Midwest	28,678	23.6
NH Black	10,436	11.2	Northeast	21,025	18.5
NH American Indian/Alaska Native	851	0.5	South	36,088	35.9
NH Asian	5,143	5.8	West	26,217	22.0
NH Hawaiian/Pacific Islander	357	0.3			
NH Multiracial	1,378	1.2			
Hispanic	11,974	14.5			
Marital status			Survey year		
Married (living with a spouse)	61,089	51.3	2010–11	58,147	49.1
Widowed, divorced or separated	20,762	16.7	2014–15	53,861	50.9
Never married	30,157	32.0			
Smoking status			Survey mode		
Smoker	16,456	14.1	Phone interview	72,377	62.7
Nonsmoker	95,552	85.9	In-person interview	39,631	37.3

*Note*. Percentages are based on the population counts, i.e., weighted counts.

**Table 2.** Workplaces with smoking restrictions and support for smoking cessation

State	All employees: percentage of employees who reported having workplace smoking restrictions		Smokers only: percentage of smokers who reported having workplace smoking restrictions		All employees: percentage of employees who reported having workplace support for smoking cessation		Smokers only: percentage of smokers who reported having workplace support for smoking cessation	
	2010–11 (%)	2014–15 (%)	2010–11 (%)	2014–15 (%)	2010–11 (%)	2014–15 (%)	2010–11 (%)	2014–15 (%)
Midwestern region								
Illinois	94.6	94.9	95.2	94.0	**21.1**	**26.6**	16.7	23.1
Indiana	**90.5**	**94.9**	89.5	88.2	**23.7**	**34.2**	19.6	23.1
Iowa	95.1	94.0	93.5	93.8	**29.1**	**35.8**	21.8	29.3
Kansas	94.3	94.8	92.9	94.7	27.3	30.1	20.7	26.1
Michigan	95.2	94.8	94.2	93.8	28.1	31.7	21.3	22.3
Minnesota	95.8	95.9	94.2	91.0	**31.3**	**41.0**	27.3	32.7
Missouri	91.7	91.6	87.2	87.9	**30.9**	**38.2**	**21.3**	**31.8**
Nebraska	**90.8**	**95.6**	91.9	88.0	**24.9**	**30.0**	24.0	23.5
North Dakota	94.6	95.9	91.7	93.7	**27.6**	**33.2**	23.8	25.9
Ohio	94.9	93.6	93.4	92.9	**31.0**	**36.3**	26.0	30.0
South Dakota	94.3	95.2	91.0	90.5	29.1	34.2	21.3	24.9
Wisconsin	95.0	94.9	93.0	95.7	39.4	40.8	29.3	36.1
Northeastern region								
Connecticut	94.9	93.5	92.0	88.5	**28.4**	**38.7**	23.6	33.1
Maine	95.7	96.5	93.8	98.2	39.4	42.8	40.4	39.2
Massachusetts	94.5	94.5	91.6	94.3	**22.3**	**30.3**	25.7	32.8
New Hampshire	94.6	93.0	93.6	89.1	**23.2**	**34.1**	17.4	27.4
New Jersey	95.0	92.7	95.2	93.2	**19.4**	**25.5**	15.0	24.9
New York	**93.9**	**91.8**	**95.1**	**88.0**	**18.6**	**25.9**	19.4	25.2
Pennsylvania	**95.2**	**92.2**	93.2	91.1	**26.7**	**33.2**	25.9	28.5
Rhode Island	95.1	93.3	90.8	93.0	27.4	32.0	22.5	27.8
Vermont	94.7	93.7	90.0	90.8	37.3	42.0	25.4	35.0
Southern region								
Alabama	95.0	94.4	90.9	92.1	**20.0**	**29.5**	**9.1**	**24.1**
Arkansas	94.6	92.7	90.3	90.1	**15.0**	**29.5**	15.8	21.2
Delaware	92.7	93.3	90.5	97.5	**19.3**	**38.3**	**17.8**	**33.4**
District of Columbia	94.5	92.6	**98.9**	**90.5**	**16.1**	**25.7**	11.2	19.5
Florida	92.2	91.3	91.7	88.6	**21.4**	**26.4**	20.4	23.0
Georgia	91.6	92.2	88.7	89.3	**17.1**	**25.8**	21.8	28.2
Kentucky	93.2	93.2	89.5	92.6	**24.7**	**33.1**	24.1	25.2
Louisiana	91.7	92.6	91.5	88.9	**13.7**	**19.6**	13.7	19.0
Maryland	94.5	93.8	91.7	90.5	28.0	27.7	17.8	17.7
Mississippi	90.0	90.0	79.3	88.8	24.0	27.6	21.4	21.2
North Carolina	93.5	94.3	90.0	94.4	**21.7**	**29.5**	18.3	21.1
Oklahoma	92.7	89.4	90.8	91.7	27.6	33.4	24.1	25.2
South Carolina	93.7	92.6	88.1	92.4	**20.9**	**27.9**	17.2	14.6
Tennessee	**96.9**	**93.6**	95.1	96.2	**22.5**	**29.7**	**17.4**	**29.1**
Texas	91.1	90.8	88.0	87.8	**17.2**	**23.2**	**10.2**	**16.3**
Virginia	94.1	92.4	91.6	90.4	**21.4**	**31.4**	**16.1**	**26.8**
West Virginia	94.8	93.3	95.2	94.3	21.9	27.2	16.4	21.8
Western region								
Alaska	92.8	94.5	86.8	93.4	28.4	33.1	17.8	27.5
Arizona	94.4	94.0	88.2	89.4	**28.1**	**36.2**	25.7	25.0
California	92.1	92.0	89.8	89.5	21.0	22.4	19.2	18.6
Colorado	94.0	93.1	90.2	90.0	**28.1**	**34.9**	25.0	27.5
Hawaii	**95.8**	**92.8**	92.2	89.9	**21.3**	**15.0**	23.6	14.0
Idaho	91.9	91.8	90.0	93.7	**23.3**	**31.6**	22.7	31.2
Montana	95.5	96.1	**99.3**	**93.5**	26.0	31.3	22.0	23.1
Nevada	89.1	89.7	89.5	89.5	25.2	22.1	18.5	21.6
New Mexico	93.1	89.6	89.0	88.9	**18.2**	**30.1**	**13.4**	**27.8**
Oregon	**98.5**	**94.4**	98.2	96.3	**33.0**	**42.5**	23.2	27.6
Utah	94.6	93.3	90.8	96.1	33.2	37.5	30.1	27.3
Washington	97.4	95.9	96.4	95.2	34.0	37.0	23.4	29.8
Wyoming	**89.1**	**93.6**	**83.6**	**91.6**	21.6	24.5	17.8	23.6
Overall	**93.6**	**92.9**	91.7	91.2	**23.7**	**29.4**	**20.2**	**24.8**

*Note*. Significant at the 5% level results (not adjusted for multiplicity) are bold.

**Table 3.** Model-assisted odds ratios and simultaneous confidence intervals for sociodemographic characteristics and smoking status

Characteristic	Workplace smoking restrictions		Workplace support for smoking cessation	
	Odds ratio	95% Confidence interval	Odds ratio	95% Confidence interval
Age (versus 65+)				
18–24	**0.79**	**(0.64–0.98)**	**0.74**	**(0.65–0.86)**
25–44	0.93	(0.78–1.11)	**1.35**	**(1.21–1.50)**
45–64	**1.23**	**(1.03–1.47)**	**1.57**	**(1.42–1.75)**
Sex (versus female)				
Male	**0.85**	**(0.80–0.90)**	**1.05**	**(1.01–1.09)**
Race/ethnicity (versus NH White)				
NH Black	**0.87**	**(0.76–0.99)**	0.93	(0.86–1.01)
NH American Indian/Alaska Native	0.64	(0.40–1.02)	0.98	(0.73–1.30)
NH Asian	**0.59**	**(0.49–0.71)**	**0.69**	**(0.62–0.77)**
NH Hawaiian/Pacific Islander	1.06	(0.43–2.63)	1.15	(0.74–1.80)
NH Multiracial	0.86	(0.61–1.21)	**1.25**	**(1.02–1.53)**
Hispanic	**0.64**	**(0.58–0.72)**	**0.67**	**(0.62–0.72)**
Marital status (versus never married)				
Married (spouse present or absent)	**1.12**	**(1.03–1.22)**	**1.21**	**(1.15–1.27)**
Widowed, divorced or separated	**1.17**	**(1.05–1.30)**	**1.18**	**(1.11–1.26)**
Highest level of education (versus above college)				
Below High school	**0.37**	**(0.31–0.45)**	**0.34**	**(0.30–0.39)**
High school or equivalent	**0.55**	**(0.48–0.64)**	**0.61**	**(0.57–0.65)**
Some or completed college	**0.72**	**(0.62–0.82)**	**0.89**	**(0.84–0.94)**
Metropolitan status of Residence (versus non-metropolitan)				
Metropolitan	1.08	(0.99–1.17)	**1.08**	**(1.02–1.15)**
Smoking status (versus nonsmoker)				
Smoker	**0.80**	**(0.73–0.87)**	**0.85**	**(0.81–0.89)**

*Note*. Significant (after adjustment for multiplicity) results are bold
