# Supplementary material for: Workplace smoking restrictions and support for smoking cessation in the USA: state, region, and overall trends from 2010–11 to 2014–15
Source: J Smok Cessat. Author manuscript; Available in PMC 2021 Mar 26. (PMC7994934; doi:10.1017/jsc.2019.10)
Supplement: 1 [file NIHMS1528893-supplement-1.docx]

Supplemental Table. State-level Sample Sizes

| **State** | **All Employees** | | **Smokers Only** | |
| --- | --- | --- | --- | --- |
|  | **2010-11** | **2014-15** | **2010-11** | **2014-15** |
| **Northeastern Region** | | | | |
| Alaska | 721 | 707 | 139 | 88 |
| Arizona | 636 | 658 | 92 | 72 |
| California | 4,440 | 3,826 | 414 | 272 |
| Colorado | 1,147 | 811 | 168 | 95 |
| Hawaii | 717 | 643 | 95 | 65 |
| Idaho | 595 | 756 | 111 | 106 |
| Montana | 464 | 1,003 | 90 | 171 |
| Nevada | 754 | 794 | 113 | 107 |
| New Mexico | 339 | 721 | 46 | 91 |
| Oregon | 846 | 694 | 123 | 84 |
| Utah | 592 | 700 | 56 | 49 |
| Washington | 1,024 | 1,013 | 138 | 138 |
| **Midwestern Region** | | | | |
| Alabama | 590 | 815 | 98 | 145 |
| Arkansas | 662 | 858 | 133 | 147 |
| Delaware | 823 | 725 | 138 | 91 |
| District of Columbia | 922 | 1,023 | 101 | 109 |
| Florida | 2,220 | 1,957 | 306 | 213 |
| Georgia | 1,222 | 1,186 | 154 | 132 |
| Kentucky | 895 | 823 | 205 | 144 |
| Louisiana | 487 | 1,009 | 87 | 155 |
| Maryland | 1,249 | 874 | 160 | 96 |
| Mississippi | 527 | 895 | 76 | 148 |
| North Carolina | 1,201 | 1,169 | 184 | 172 |
| Oklahoma | 749 | 642 | 164 | 88 |
| South Carolina | 804 | 840 | 143 | 117 |
| Tennessee | 721 | 896 | 142 | 141 |
| Texas | 2,968 | 2,953 | 425 | 369 |
| Virginia | 1,114 | 1,042 | 147 | 143 |
| West Virginia | 502 | 725 | 107 | 133 |
| **Southern Region** | | | | |
| Illinois | 1,842 | 1,658 | 324 | 218 |
| Indiana | 995 | 968 | 175 | 184 |
| Iowa | 1,194 | 832 | 232 | 126 |
| Kansas | 1,077 | 819 | 186 | 141 |
| Michigan | 1,430 | 1,284 | 253 | 220 |
| Minnesota | 1,677 | 1,202 | 280 | 175 |
| Missouri | 948 | 843 | 199 | 154 |
| Nebraska | 1,065 | 898 | 178 | 135 |
| North Dakota | 987 | 824 | 157 | 156 |
| Ohio | 1,799 | 1,632 | 367 | 296 |
| South Dakota | 1,150 | 831 | 243 | 162 |
| Wisconsin | 1,556 | 1,167 | 256 | 201 |
| **Western Region** | | | | |
| Connecticut | 1,315 | 805 | 194 | 93 |
| Maine | 983 | 747 | 158 | 110 |
| Massachusetts | 994 | 999 | 114 | 106 |
| New Hampshire | 1,158 | 849 | 172 | 121 |
| New Jersey | 1,108 | 914 | 120 | 88 |
| New York | 2,398 | 1,994 | 317 | 233 |
| Pennsylvania | 1,917 | 1,576 | 338 | 241 |
| Rhode Island | 881 | 605 | 143 | 61 |
| Vermont | 944 | 838 | 144 | 125 |
| Wyoming | 798 | 818 | 256 | 201 |
|  |  |  |  |  |
| **Overall** | 58,147 | 53,861 | 9,068 | 7,388 |
